# Supplementary material for: Organic Electrochemical Transistors as Versatile Tool for Real-Time and Automatized Viral Cytopathic Effect Evaluation
Source: Viruses. 2022 May 26;14(6):1155. doi: 10.3390/v14061155 (PMC9227436; doi:10.3390/v14061155)
Supplement: Supplementary file 1 [file viruses-14-01155-s001.zip › viruses-1729921-supplementary.pdf]

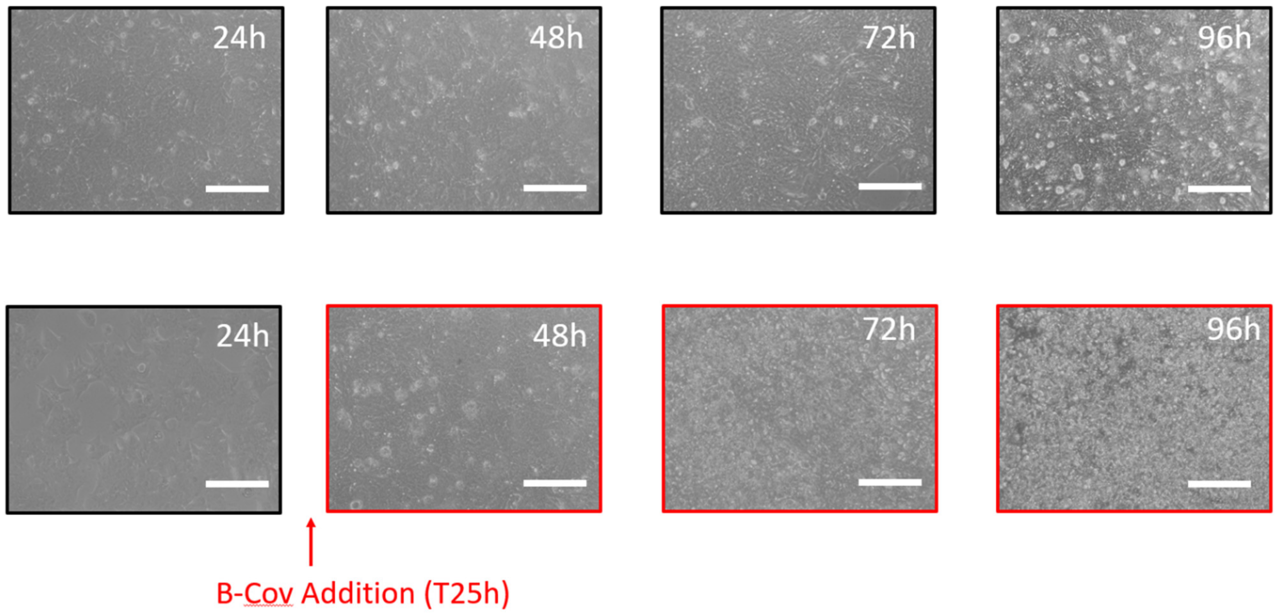

Figure S1. Optical micrographs of HRT-18 cells, grown over time in standard conditions (black border, top line) and after the infection with Bovine Corona virus at 25h post seeding (red border, bottom line). White scale bar = 150 μm.

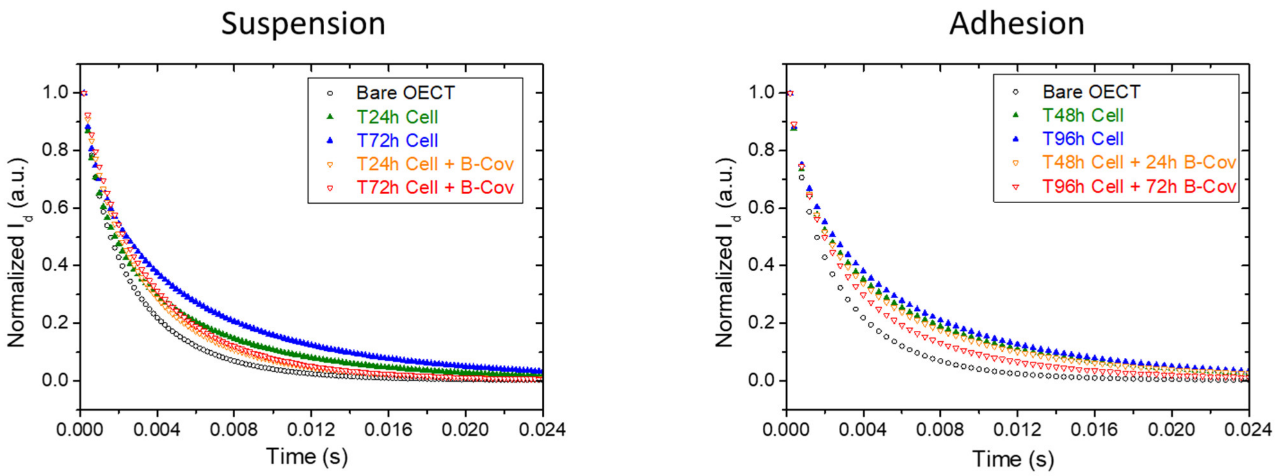

Figure S2. Normalized current modulation upon the application of a voltage potential on the gate before seeding (black empty circles), during HRT-18 healthy cell growth at 24h (green filled triangles) and 72h (blue filled triangles) and after 24h and 72h of B-Cov infection.

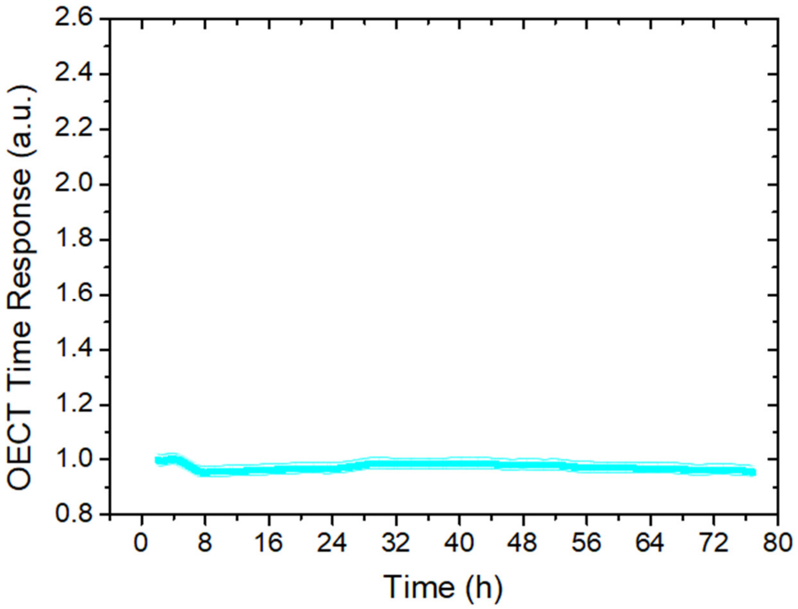

Figure S3. Electrical control (without cells) of the OECT, incubated with Bovine Corona Virus for 72h.

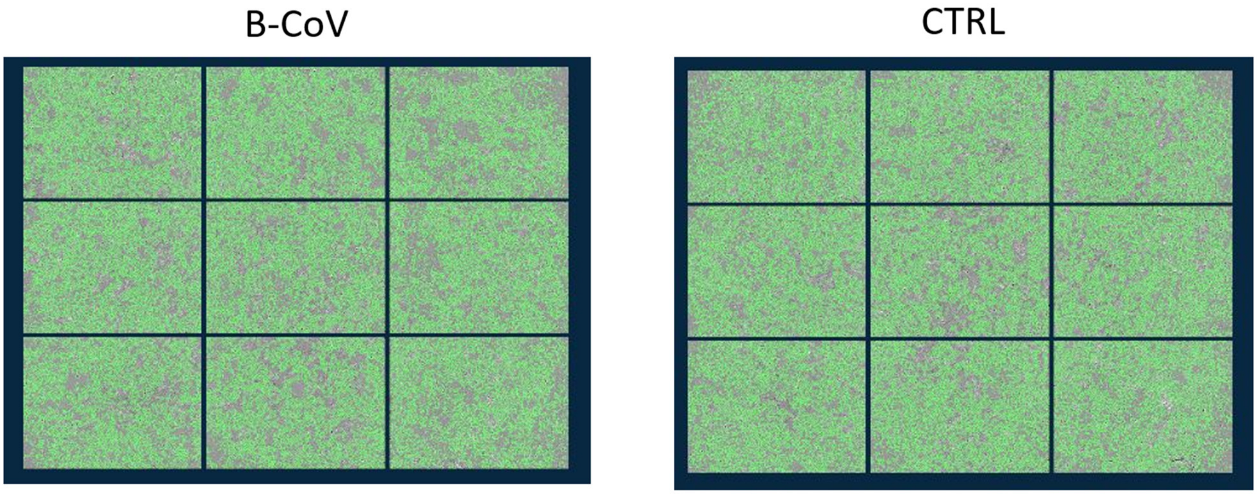

Figure S4. Multi-imaging reconstruction taken by the optical-based technology Incucyte® of HRT-18 cells, infected by B-CoV (on the left) and healthy grown (control, CTRL, on the right) after 48h.

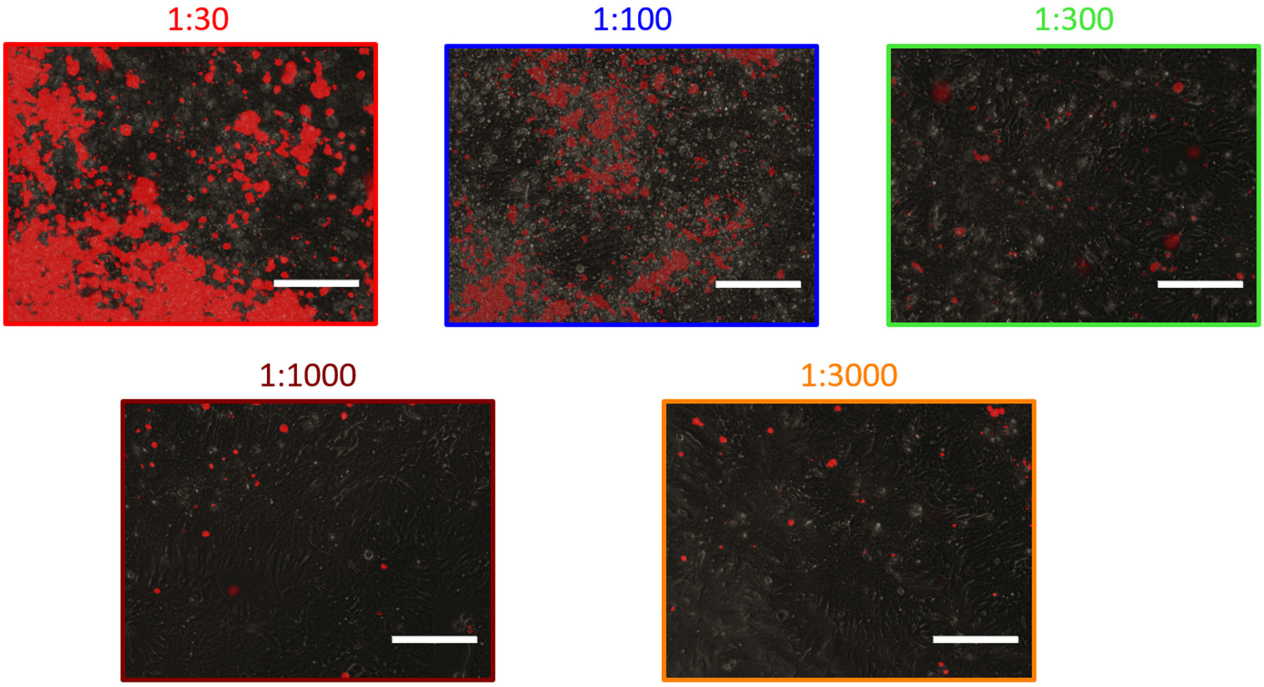

Figure S5. Optical micrographs on HRT-18, infected with progressive dilutions of B-Cov-after 72h (in suspension), using a red dye staining for the dead cells. White scale bar = 150 μm.

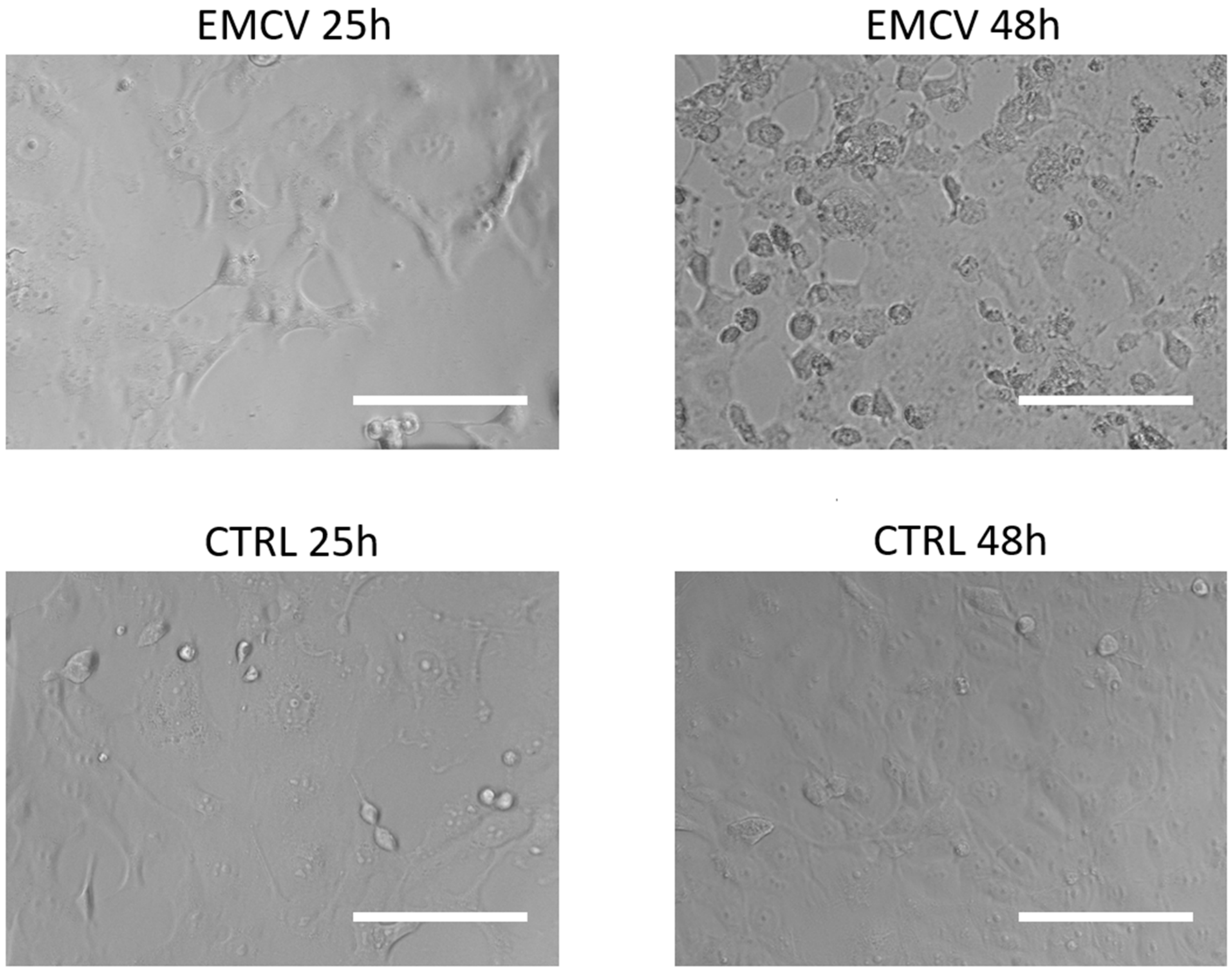

Figura S6. Optical micrographs of VERO E6 after 25h and 48h of EMCV infection (top left and right, respectively), compared with VERO E6 standard growth (control, bottom left and right, respectively). White scale bars = 150 μm.

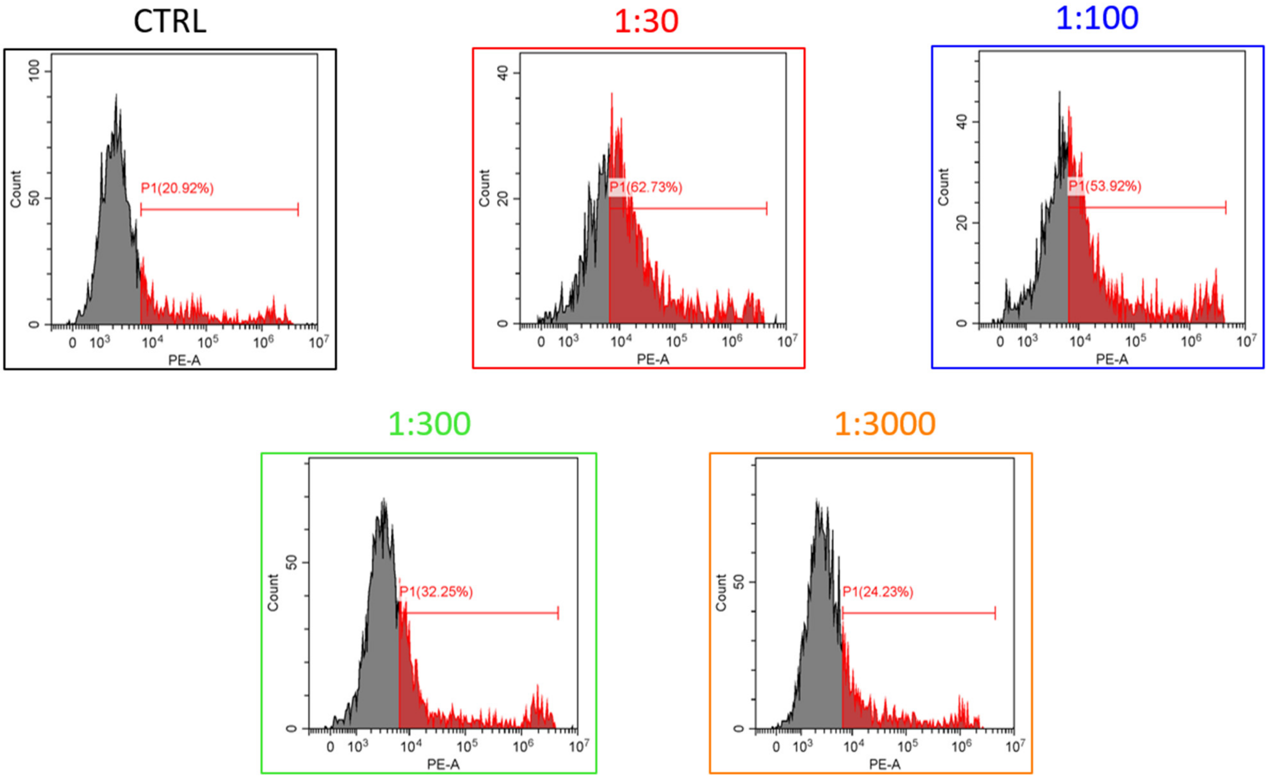

Figura S7. Cytofluorimeter analysis on VERO E6, infected with progressive dilutions of EMCV, after 48h of incubation, compared with a standard growth (CTRL).

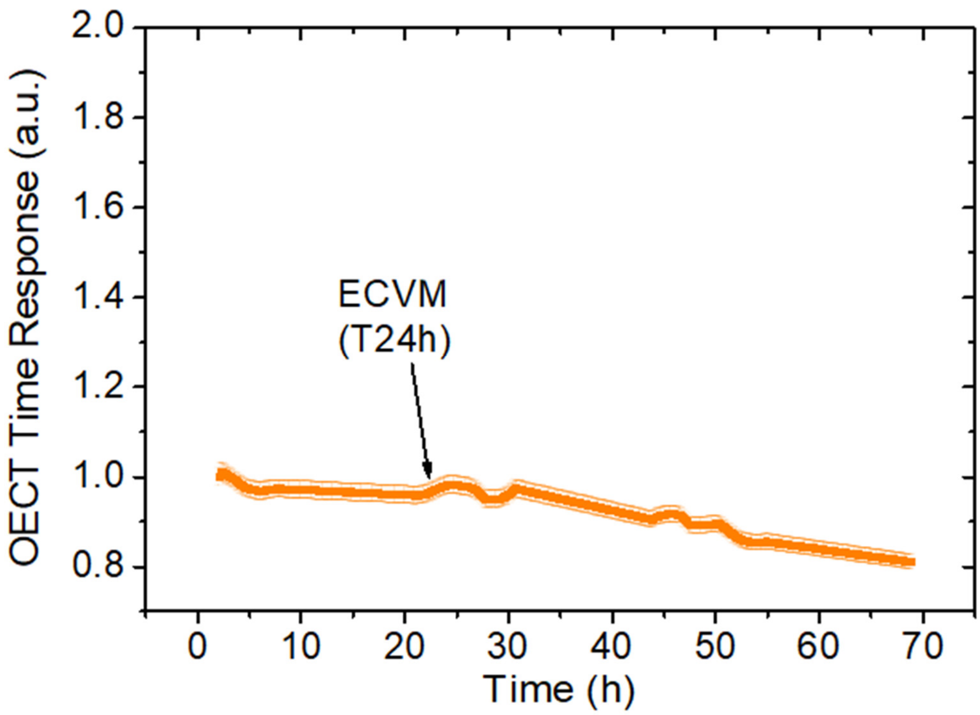

Figure S8. Electrical control (without cells) of the OECT, incubated with EncephaloMyocarditis Virus after 24h from the beginning of the experiment, mimicking the biological one (in adhesion).
